# Synthesis, characterization, and biodegradation studies of new cellulose-based polymers

**DOI:** 10.1038/s41598-023-28298-5

**Published:** 2023-01-30

**Authors:** F. E. Tabaght, K. Azzaoui, A. El Idrissi, S. Jodeh, B. Khalaf, L. Rhazi, R. Bellaouchi, A. Asehraou, B. Hammouti, R. Sabbahi

**Affiliations:** 1 Laboratory of Applied Chemistry and Environment, Faculty of Sciences, Mohammed First University, 60000 Oujda, Morocco; 2 Laboratory of Engineering, Electrochemistry, Modeling and Environment, Faculty of Sciences, Sidi Mohamed Ben Abdellah University, 30000 Fez, Morocco; 3 Department of Chemistry, An-Najah National University, P.O. Box 7, Nablus, Palestine; 4 Department of Chemistry, Arab American University, Jenin, Palestine; 5 Institut Polytechnique UniLaSalle Transformations & Agro-Resources Research Unit (ULR7519), 19 Rue Pierre Waguet, BP 30313, 60026 Beauvais, France; 6 Laboratory of Biochemistry and Biotechnology, Mohammed First University, Faculty of Sciences, 60000 Oujda, Morocco; 7 Laboratory of Development and Valorization of Resources in Desert Zones, Higher School of Technology, Ibn Zohr University, Quartier 25 Mars, P.O. Box 3007, Laayoune, Morocco

**Keywords:** Environmental sciences, Chemistry, Materials science, Nanoscience and technology

## Abstract

New cellulose carbamates and cellulose acetate carbamates were prepared by classical addition reaction of isocyanates with alcohols. A Telomerization technique was used to make the grafted molecules strongly anchored and more hydrophobic. These molecules were grafted into cellulose and CA chains, respectively. The structures of the synthesized derivatives were confirmed using Nuclear Magnetic Resonance Spectroscopy, Fourier Transform Infrared and Thermogravimetric Analysis, and their solubility phenomenon was also established, and the carbamate derivatives showed better solubility compared to cellulose. Their ability to biodegrade was investigated, and it was concluded that Cell-P_1_ and CA-P_1_ derivatives are more biodegradable than the other samples. These results suggest that the resulting compounds can be used effectively in many useful industrial fields, for instance, eco-friendly food packaging, domains that use materials that are environmentally friendly and sustainable and the development of green chemistry.

## Introduction

In this period of having emergent necessity for natural resources preservation and environmental protection, as a result of global concerns about hygiene and health matters. Continuous research and development in the realm of sustainable and ecologically friendly compounds are exponentially important for current generations and even more vital so for future generations^1^. Cellulose is considered to be the dominant constituent present in the vast majority of plants^2^. It has specific characteristics such as, regular structure, renewable resources, biodegradability, and abundance. Cellulose structure and characteristics offer unique and outstanding options for systematic projects in order to develop appropriate substances for many relevant fields, including but not limited to, membrane technology^3–5^, pharmaceutical applications^6–8^, removal of organic dyes, toxic heavy metals, anions and cations from groundwater and other aqueous solutions^9–12^, bioelectronics^13^ and other industries, for example; food, textile, and paper^14^. Cellulose exploitation represents a very rapidly growing sector that includes the high-volume commodities industry, for instance, paper and textiles, as well as, the production of novel high-added-value materials, for example; functionalized fibers and natural reinforcing elements that exist in fiber-based composite substances^15^.

Cellulose represents a multifunctional biological material with a high molar mass. In order to improve its processing ability, precursors are used for providing diverse varieties of cellulose derivatives^2^. The cellulose chemical modification was performed on the reactive hydroxyl groups as various processes can be carried out, resulting in a broad variety of novel commercial substances^16,17^.

Being the most abundant polymer in nature does not mean it’s easy to handle. The presence of inter- and intramolecular hydrogen bonds in cellulose can result in the lack of its processability and solubility. One of the significant processes, in order to make this polymer a highly processable one, is the preparation of cellulose acetate such that hydroxyl groups will be converted into acetates. There are other cellulose modification achievements that were reported, such as; esterification^18^, etherification^19^, oxidation^2^, etc. Chemical modification of cellulose with isocyanate or blocked diisocyanate groups has been largely investigated^17^. It was found that this modification has such unique features as a relatively high rate, the absence of secondary products, and the chemical stability of the urethane moieties^20^. In addition, the blocked isocyanate functions are also widely used and patented for different applications, such as; crosslinking and coating adhesives of solid propellants, as well as, they have many advantages compared to the isocyanates^21^. Different types of isocyanate and blocked diisocyanate groups have been used to modify cellulose, and the carbamate derivatives resulting from this modification have been developed for several applications, for example; gas permeation^2^, membrane materials^21,22^, film materials in hemodialysis fields^23^, reinforcement in composites^17,24^, and chiral stationary phases^25^. Various researchers have studied synthesis of the cellulose carbamate and the cellulose carbamate derivatives^26–28^.They can be prepared using multiple synthesis routes. Cellulose carbamate in conventional processes, is produced by reaction with urea in a basic or an acidic medium^29–33^. This reaction required high temperature values and long contact time. Other methods for cellulose carbamate synthesis have been reported including, microwave heating^34,35^, electron radiation^36–38^, supercritical CO_2_ assisted impregnation^39–41^, and liquid–solid phase technique^42^.

To our knowledge, the preparation procedure and the characteristics of the novel cellulose carbamate and cellulose acetate carbamate were not deeply described. In this research, the chemical modification of the cellulose extracted from the esparto grass, *Stipa tenacisima*, of Eastern Morocco using blocked isocyanate compounds, was studied in order to prepare carbamate derivatives of cellulose and cellulose acetate. A telomerization technique is used to prepare a precursor which is attached to cellulose using a grafting procedure. Moreover, this synthesis method is eco-friendly, taking into account some green chemistry principles (synthesis in one pot, any residues or wastes, and pollution-free) and can easily lead to high DS. The samples obtained are characterized using several spectroscopic methods, for instance; FT-IR and NMR spectroscopies. Their chemical structures were investigated, as well as, the physical properties such as thermal stability, crystallinity, solubility, etc., were studies. The carbamate derivatives were also submitted for biodegradation tests.

It is important to emphasize that the plant (*S. tenacissima*) used in this work is wild, it has been used for a long time to make handicrafts. In addition to previously mentioned, the purpose of our research is to further enhance this plant. We note that this plant is renewable each year and it must be cultivated so that it grows back better. Therefore, we declare that the collection and use of this plant in the field of research is in accordance with national guidelines and legislation. The plant material used in this study was identified by El Idrissi et al*.*^43^. A voucher specimen was stored at Laboratory of Applied Chemistry and Environment, Mohammed first University, Faculty of Sciences, Oujda, Morocco.

## Materials and methods

### Chemicals and reagents

Lithium chloride (LiCl 98%) was obtained from Riedl-de Haën (Germany). Dibutyltindilaurate (DBTDL 95%) and 2, 2′-Azobisisobutyronitrile (AIBN) were obtained from Aldrich and used as catalysts and initiator respectively. N,N-dimethylacetamide (DMA, ≥ 99%), hexamethylene diisocyanate (HDI, 98%), 1,6-hexamethylene dodecanethiol (≥ 98%), butan-1-ol and undec-10-enol were purchased from Sigma-Aldrich (USA). The other chemical materials were of analytical grade, and have been used without any further purification. Lixivia was recuperated from landfill site of Oujda city (Morocco).

### Cellulose extraction

Cellulose (M_w_ $\approx$ 227,200 g/mol & DP_w_ ≈ 1402) was extracted from *Stipa tenacissima*, of Eastern Morocco using the chemical process developed by El Idrissi et al*.*^43^. Isolated cellulose sample was dried at 90 °C in a vacuum oven to constant weight and ground into a fine powder, which was additionally dried before use.

### FT-IR analysis

Shimadzu Fourier transform infrared spectrometer FT-IR-8400S was used for the prepared cellulose derivatives; for this analysis, a ${\mathrm{KB}}_{\mathrm{r}}$ disks containing 2% of finely dispersed samples were prepared. Twenty scans were done of each sample, recorded from 4000 to 400 cm^−1^ with a resolution of 4 cm^−1^.

### NMR analysis

Proton Nuclear Magnetic Resonance spectra for the synthesized cellulose derivatives were recorded using a Bruker spectrometer (300 MHz) at the National Center for Scientific and Technical Research Morocco (CNRST) at Rabat, Morocco. Deuterated dimethyl sulfoxide (DMSO) and dichloromethane-d2 (CD_2_Cl_2_) were used as solvents. Tetramethylsilane (TMS) has been used as the internal standard. All H^1^ NMR spectra were recorded using 16 scans with a pulse delay of twenty seconds.

### Thermal analysis (TGA/TDA)

Thermal stability of cellulose derivatives was determined using a thermogravimetric analyzer (TGA, Q500) from TA instruments. 10–15 mg of samples were placed in an aluminum pan, and the operation was done in a nitrogen atmosphere (50 ml/min). Each sample was heated from 25 to 700 °C at a rate of 15 °C min^−1^. The mass loss for each sample at different temperature values was recorded.

### X-ray diffraction (XRD)

The X-ray diffraction (XRD) of the samples was performed using a diffractometer Shimdza XRD 6000 with CuKα-radiation (λ = 0.154 Å). The ordering index (OI) was estimated by the Eq. (1):^44^

1 $$OI \left(\%\right)=\frac{(I\_22-I\_18)}{I\_22 }*100$$

where I18 represents the intensity of diffraction at 2θ = 18°, while I22 represents the intensity of diffraction at 2θ = 22°.

### Preparation of cellulose solution

According to the procedure of El Idrissi et al*.*^43^, 0.292 g of cellulose and 25 mL of DMAc were placed in a three-necked round-bottom flask equipped with a thermometer, magnetic stirrer and reflux condenser. The mixture was heated at 150 °C for 30 min, then cooled to 100 °C and 9% (3 g) of LiCl was added. Then the mixture was cooled to 50 °C and stirred for 15 h until a clear cellulose solution was obtained.

### Synthesis of cellulose carbamates (Cell-P_1_ and Cell-P_2_)

First, a telomere was prepared using telomerization reaction. In a three-necked round-bottom flask that was equipped with an addition funnel, magnetic stirrer, thermometer, and a reflux condenser, 1 mmol of dodecanethiol was introduced. 1 mmol of undec-10-enol containing 10^–2^ mmol of AIBN catalyst was introduced in a funnel and added dropwise into dodecanethiol. The system was kept at 80 °C in a nitrogen atmosphere under stirring for 3 h. In another three-necked round-bottom flask that was equipped with a thermometer, addition funnel, reflux condenser and magnetic stirrer, 1.05 mmol of (1,6)-HDI was introduced. The OH-containing telomere prepared at the first stage, was mixed with small amount of DBTDL catalyst, introduced in a funnel, and then added dropwise to 1,6-HDI in the round-bottom flask. The reaction mixture was maintained for 3 h at 80 °C, and kept under stirring in a nitrogen atmosphere. The end product was a blocked isocyanate of fairly high yield. Finally, cellulose carbamate (Cell-P_1_) was synthesized according to the method by El Idrissi et al*.*^43^. For this purpose, the obtained blocked isocyanate was added to the cellulose solution containing small amount of DBTDL catalyst and the mixture was kept under stirring at 80 °C for 3 h in a nitrogen atmosphere. The resulting product (Cell-P_1_) was collected after precipitation with a mixture of methanol/water (1/3). Then this product was isolated by filtration using a membrane filter, washed successively with distilled water, methanol, and then dried under vacuum at 80°C^22^. The cellulose carbamate named (Cell-P_2_) was also prepared and isolated using the same procedure as (Cell-P_1_), but using butan-1-ol as a short alcohol to prepare another precursor.

### Preparation of cellulose acetate (CA)

Cellulose acetate was prepared according to the method described in the literature^45^. Briefly, 10 g of cellulose powder was placed in a round bottom flask, with 80 mL of acetic acid, 120 mL of toluene and 2 mL of perchloric acid. The mixture was vigorously shaken for a few minutes and then 50 mL of acetic anhydride was added. The reaction was stopped after 10 min. Finally, cellulose acetate (DS = 1.7) was isolated by precipitation in distilled water and dried overnight in an oven at 90 °C.

### Synthesis of cellulose acetate carbamates (CA-P_1_ and CA-P_2_)

In a three-necked round-bottomed flask equipped with a magnetic stirrer, addition funnel, thermometer and reflux condenser, 1.05 mmol of 1,6-HDI was introduced. Then 1 mmol of the already prepared telomere was mixed with a small amount of DBTDL catalyst, placed in a funnel and added dropwise to 1,6-HDI. The reaction system was kept at 80 °C for 3 h under a nitrogen atmosphere. Thereafter, an amount of cellulose acetate (0.4 g) solubilized in DMSO was added to the reaction system. The resulting product (CA-P_1_) was precipitated with methanol, filtered, washed with methanol and distilled water, and then dried in an oven for a day at 60 °C. The CA carbamate (CA-P_2_) was also synthesized and isolated using the same method as stated above, where the precursor was prepared by reaction of 1,6-HDI with butan-1-ol.

## Biodegradation study

The samples tested for biodegradability are ground powders having dimensions in the order of microns.

### Growth microorganisms on the polymer samples

The biodegradation under aerobic conditions was conducted on culture medium and adopted in the same form that was recommended by German Institute of Standardization (DIN 53739), American Society for Testing and Materials (ASTM G21-70, G22-76, G29-75), French Association for normalization (AFNOR X 41-514-81 and X41-517-69), and the International Organization for Standardization (ISO 846)^46^. Briefly, the tested cellulose derivatives were placed on the culture medium (M_S_) surface as the sole carbon source. The culture medium was consisted of 0.7 g monopotassium phosphate (KH_2_PO_4_), 0.7 g of potassium hydrogen phosphate (K_2_HPO_4_), 1 g of ammonium nitrate (NH_4_NO_3_), 0.7 g of magnesium sulfate heptahydrate (MgSO_4_.7H_2_O), 0.005 g of sodium chloride (NaCl), 0.002 g of zinc sulfate heptahydrate (ZnSO_4_/7H_2_O), 0.002 g ferrous sulfate heptahydrate (FeSO_4_/7H_2_O), and 15 g of agar and 0.001 g of manganese Sulfate heptahydrate (MnSO_4_/7H_2_O), dissolved in 1 L distilled water while pH value of the culture medium was 6–6.5. The culture medium was autoclaved for 20 min at 121 °C, and then poured into sterile petri dishes. The tested polymers were placed to culture medium and sprayed with lixivia. Then, the inoculated culture media containing polymer samples were incubated at 25 °C for 28 days. After that, the tested samples were subjected to a visual assessment for growth of microorganisms.

### Determination of biochemical oxygen demand

Biodegradability assessment of the samples prepared was performed using standard test method ISO 14851^47^. The 600 mL glass flasks were filled with liquid medium to 400 mL, and kept closed with glass caps. The liquid medium that was observed represents the ‘‘standard test medium’’ according to ISO 14,851 for ultimate aerobic biodegradability measuring for plastic substances in aqueous medium technique by oxygen demand determination in a closed respirometer. The medium was based on the following solutions: Solution A consists of 21.75 g/L of K_2_HPO_4_; 8.5 g/L of KH_2_PO_4_; 0.5 g/L of NH_4_Cl; 33.4 g/L of Na_2_HPO_4_.2H_2_O, Solution B consists of 22.5 g/L of MgSO_4_.7H_2_O, Solution C consists of 36.4 g/L of CaCl_2_.2H_2_O, and Solution D consists of 0.25 g/L of FeCl_3_.6H_2_O.

To prepare the test medium, 10 ml of solution A was added to 1 mL of each solution A, B, and C, and then this volume was adjusted to 1000 mL with distilled water. A soil sample was taken to prepare the inoculum solution. Then this sample was diluted with distilled water to the final solid concentration of 200 g/L and aerated by stirring for 4 h. After that, each vessel was filled with the test medium (380 mL), and inoculated with inoculum solution (20 mL). Then the tested polymer sample, 50 mg, was added to the vessel. The blank, containing the culture medium without sample addition, was inoculated under the same conditions as the test polymer samples. Two replicates were done for the blanks and for each sample. The tests were stopped when consumption of Oxygen was not detectable anymore, in all cases, at least after 40 days at 25 °C. The OxiTop system is used for biochemical oxygen demand (BOD) determination. This system consisted of test vessels (reactors) with carbon dioxide traps (NaOH) in the headspace. These bottles are sealed with a cap that containing electronic pressure indicator, and supplied with magnetic stirrer.

The net BOD of the measured samples was determined using Eq. (2), as it represents the difference between consumption of oxygen O_2_ in the test and in the blank flasks.

2 $$net BOD={BOD}_{tm}-{BOD}_{b}$$

where BOD_b_ represents the biochemical oxygen demand of the blanks (average value of two flasks), BOD_tm_ represents the total biochemical oxygen demand for tested samples (from one flask); The biodegradation D_tm_ percentage can be determined by measuring the ratio of (*net BOD*) net biochemical oxygen demand to (total ThOD) total theoretical oxygen demand, according to the amount of tested samples that is originally conducted in the flask by using Eq. (3):

3 $${D}_{tm}=\frac{net BOD}{total ThOD}\times 100$$

where D_tm_ represents the biodegradation percentage; net BOD represents the net biochemical oxygen demand (mg O_2_/L); ThOD represents the theoretical oxygen demand (mg O_2_/L).

The biochemical oxygen demand of the studied biopolymers was investigated for a period of 40 days at 25 °C. The experiments were repeated two times. In both experiments, after this period, consumption of O_2_ in respirometers was not detectable anymore.

## Results and discussion

### Modification of cellulose and cellulose acetate

**Figure 1:**
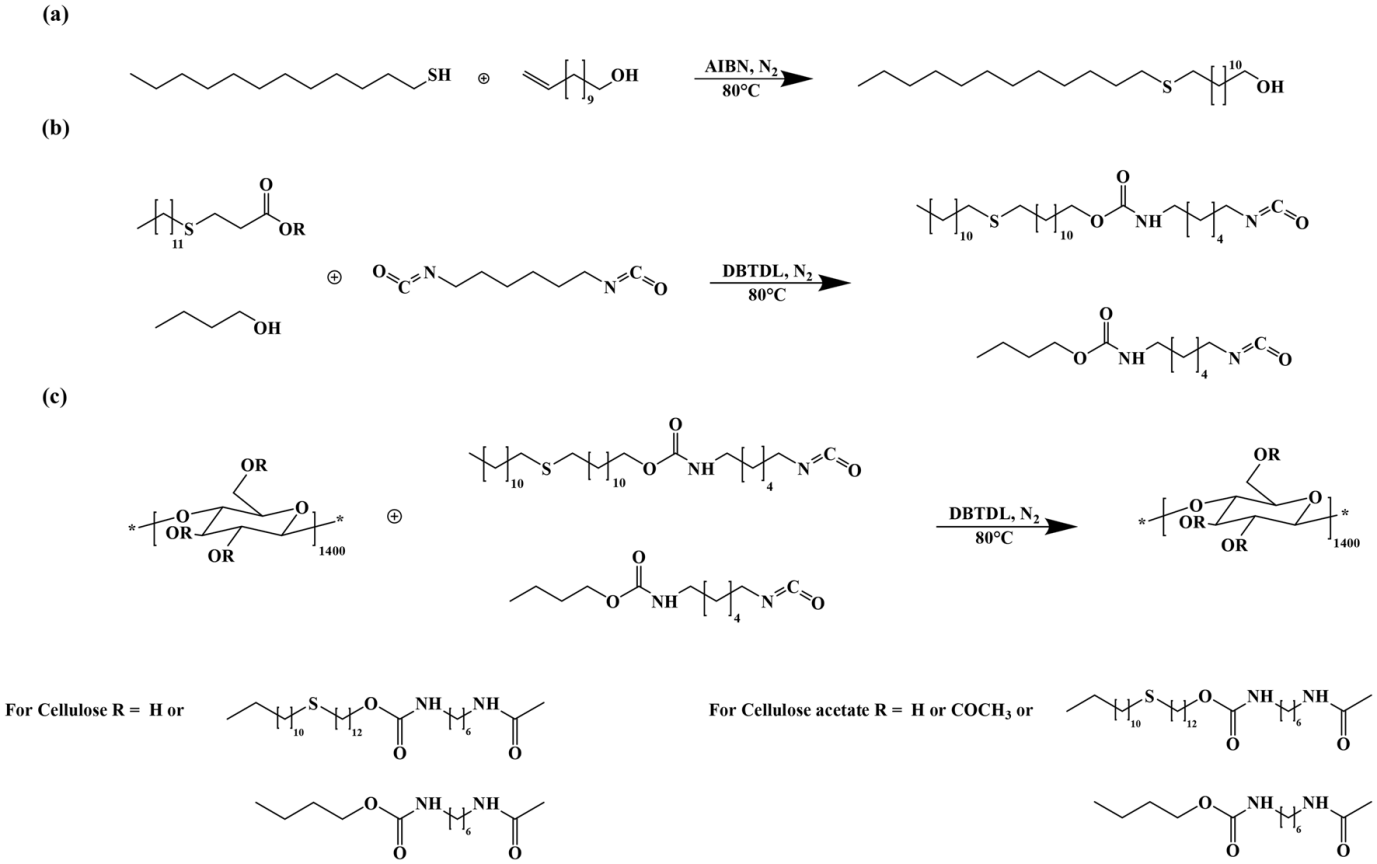
Synthesis of the: (**a**) telomere (R-S-R′-OH), (**b**) precursors (HDI-OH-RSR’ and HDI-but-OH), and (**c**) of Cell-P_1_, Cell-P_2,_ CA-P_1_ and CA-P_2_.

Cellulose and CA modification were conducted using a classical addition reaction of isocyanate with alcohol catalyzed with DBTDL. This modification is schematized in Fig. 1 and was done by 3 steps. In the first step, the thiol compound chain is lengthened by a reacting dodecanethiol and undec-10-enol using telomerization reaction (Fig. 1a). It has been demonstrated that in this thiol-ene addition, the dodecanethiol is considered as telogen, undec-10-enol as taxogen, and AIBN as an initiator leading to an adduct as reported in the literature^48^. The thiol-ene addition reaction is conducted in stoichiometric conditions, in a free solvent system, and the reaction progress is followed by the FT-IR technique. The absorbance bands designated to the –C=C– and SH groups at 1638 cm^−1^ and 2574 cm^−1^, respectively, despaired, indicating that this addition reaction is totally accomplished. For the second step, a blocked isocyanate compound is prepared by a reaction between one of the functional groups for the (1, 6)-HDI (Fig. 1b) with the alcohol group of the telomere that was already prepared. The FT-IR spectrum of this precursor shows a new sharp peak assigned to the NCO group situated at its end that was at around 2270 cm^−1^. The last step is cellulose and CA grafting, respectively with the blocked isocyanate compound that was already described in (Fig. 1c). This procedure has some advantages; firstly, the synthesis is realized scarcely in one-pot, employing a catalyst and avoiding non-stoichiometric reagent balance, leading to high selectivity. Secondly, a high degree of substitution (DS) can be reached easily. Moreover, these synthesis methods do not result in any wastes or residues, taking into account the respect of some green chemistry principles. The samples obtained are characterized by various spectroscopy methods, for instance, by FT-IR and NMR spectroscopy to investigate chemical structures, and physical properties; for example; structural order, thermal properties, solubility, etc.

### FT-IR analysis

It was noted that the properties of cellulosic material depend greatly on the interactions within and between cellulose chains. For investigating the changes that occur due to chemical modifications, Fourier Transform Infrared Spectroscopy technique (FT-IR) is usually carried out. Summarizing the observations, the researchers concluded that cellulose has mainly C-H and CH_2_ groups stretching (2700–3000 cm^−1^), hydroxyl groups (3200–3600 cm^−1^), C–O stretching (1000–1300 cm^−1^), glycosidic linkages (∼895 cm^−1^), and C–O–C stretching (∼1164 cm^−1^)^49^.

As a result of grafting the blocked isocyanate (R-NCO) to cellulose and CA, respectively, the NCO peak observed in the FT-IR spectra at 2270 cm^−1^ disappeared, which means that the NCO groups were transformed to carbamate groups. Thus, the analysis of the FT-IR spectrum for –C=O and –NH groups is very promising for studying the formation of urethanes (carbamates)^50^.

**Figure 2:**
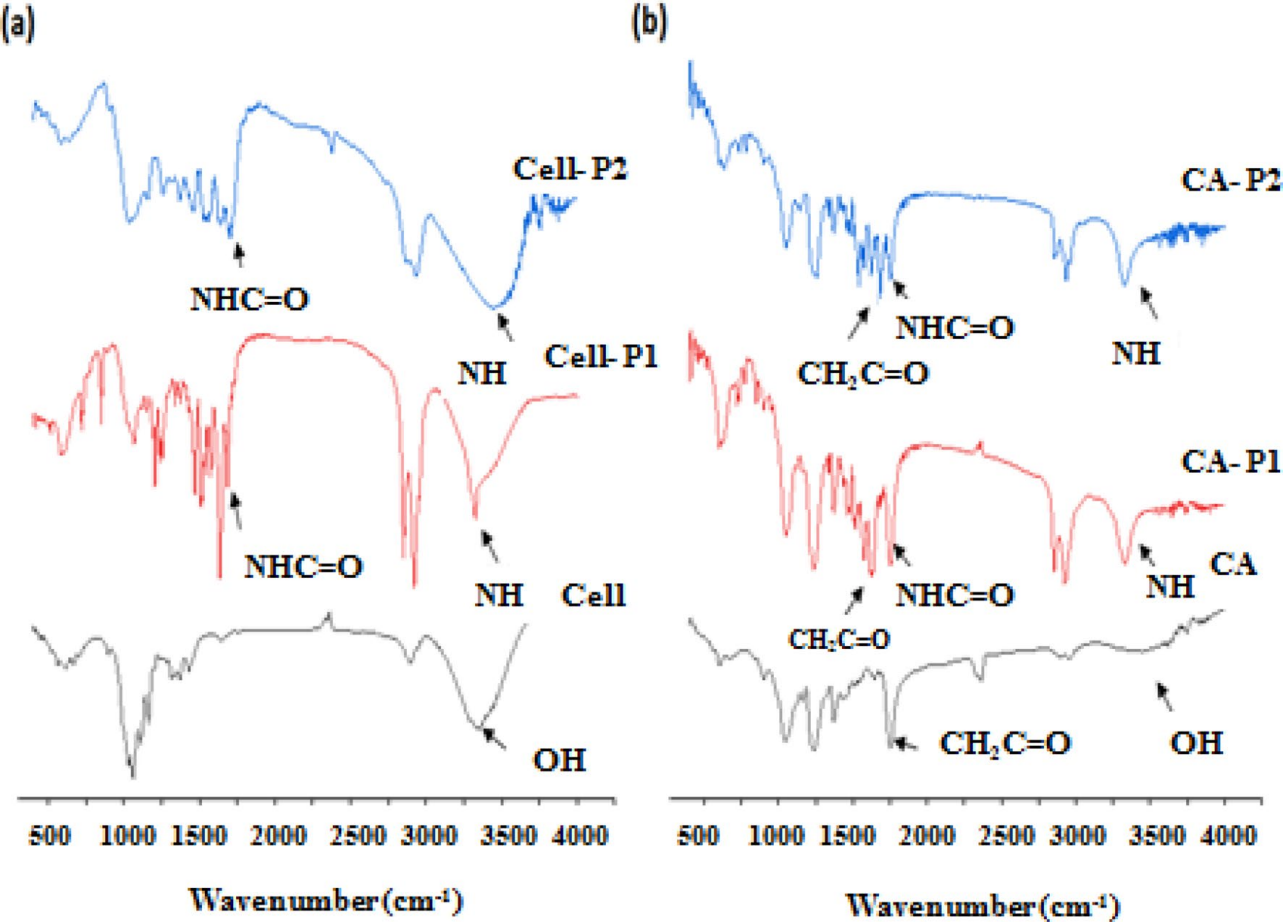
FT-IR spectra for: (**a**) cellulose, Cell-P_1_ & Cell-P_2_; and (**b**) CA, CA-P_1_, & CA-P_2_.

Figure 2 shows FT-IR spectra for cellulose, CA, and their carbamates in the range of 4000–400 cm^−1^. If the spectra of carbamates are compared with the spectrum of cellulose, then it can be concluded that for cellulose carbamates new absorption bands are observed, at 1684 cm^−1^ for Cell-P_1_ and at 1713 cm^−1^ for Cell-P_2_ (Fig. 2a). The absorption bands appearing at 3327 cm^−1^ for Cell-P_1_ and 3454 cm^−1^ for Cell-P_2_ (Fig. 2a) can be attributed to stretching vibrations of the NH groups bonded with the ether oxygen groups of cellulose. In addition, the bending vibrations of NH groups give an absorption band around 1520 cm^−1^. The intensity of the broad band at 3396 cm^−1^ of OH groups cellulose decreases significantly after the formation of carbamates. The FT-IR results confirm the conclusions by *Gironès *et al*.*^51^and *Gallego *et al*.*^52^.

The CA carbamate derivatives are also monitored by analyzing their FT-IR spectra, presented in Fig. 2b. Table 1 lists the most important peaks detected during this chemical modification.

**Table 1:** FT-IR absorption bands in CA carbamate (CA-P_1_, CA-P_2_).

Functional groups	Wave number (cm^−1^)	
	CA-P_1_	CA-P_2_
NH (stretching vibration)	3329	3325
CH/CH_2_ (stretching vibration)	2853 and 2924	2856 and 2935
HN—C=O (stretching vibration)	1749	1749
CH_3_C=O (stretching vibration)	1755	1757
C—N (stretching vibration)	1236	1238
N—H (bending vibration)	1519	1539

As can be seen, the new C=O stretching vibration band at about 1749 cm^−1^ is present in the spectrum of CA-P_1_ and CA-P_2_. This band evidences the formation of CA carbamate obtained by reacting the free isocyanate group with the remaining hydroxyl group of the CA. The decrease in the intensity of the absorption band at 1751 cm^−1^ also indicates the existence of carbamate groups in CA-P_1_ and CA-P_2_ products (Fig. 2b). The absorption bands around 2850 and 2930 cm^−1^ are assigned to stretching vibrations of CH/CH_2_ groups and their intensity increase by the addition of more CH/CH_2_ groups contained in the side chains of the grafted compounds. The NH groups bonded with carbonyl groups and/or ether oxygen groups of cellulose may be responsible for the absorption of the vibration bands appearing at 3329 and 3325 cm^−1^ for CA-P_1_ and CA-P_2_, respectively. These findings support the idea that carbamate moieties-containing graft compounds are formed via an addition reaction between alcohols and isocyanate groups.

### NMR analysis

**Figure 3:**
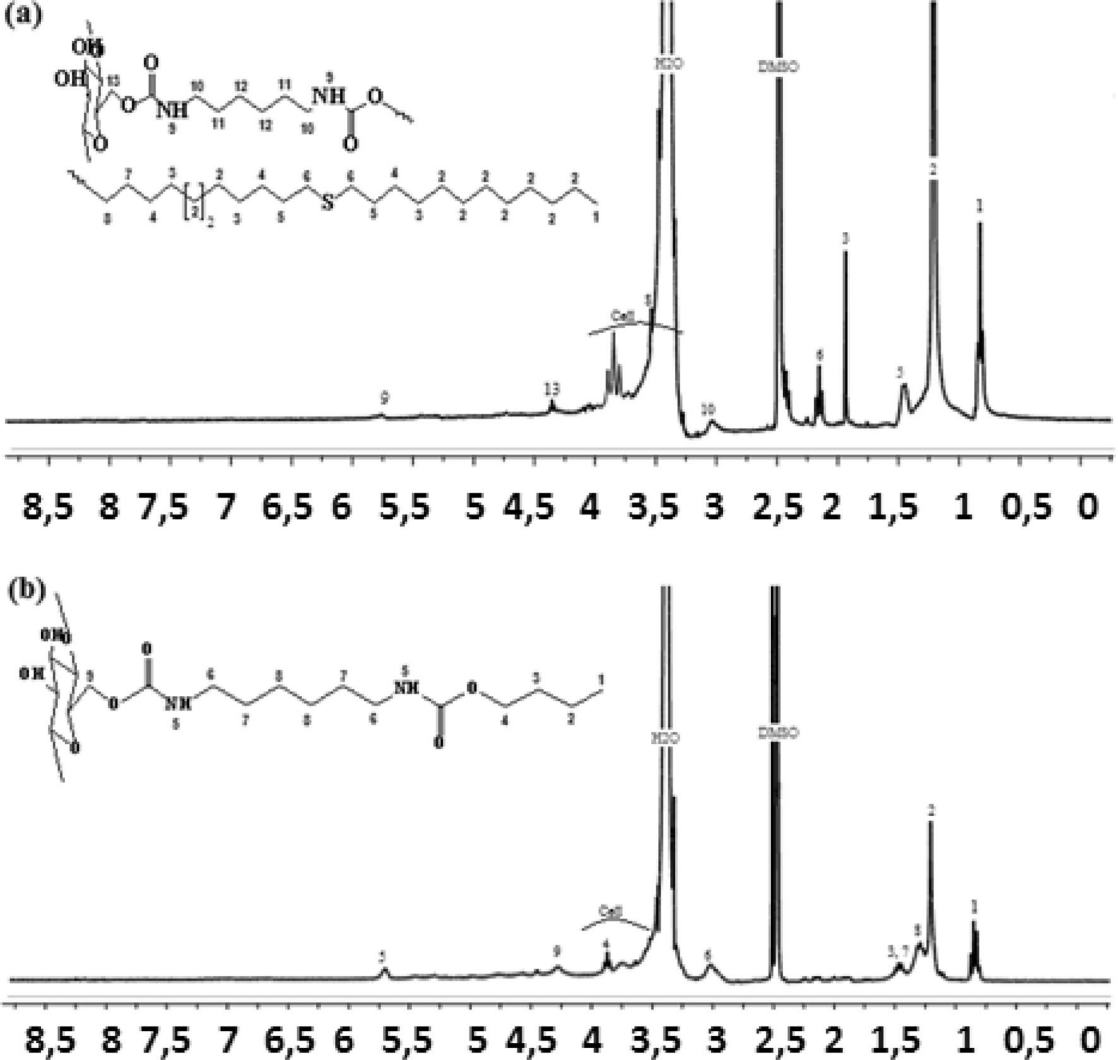
Proton NMR spectra for (**a**) Cell-P_1_ and (**b**) Cell-P_2_.

The Proton Nuclear Magnetic Resonance spectra also agree with the proposed chemical structures. Such that, ^1^H NMR spectra of cellulose carbamate (Cell-P_1_ and Cell-P_2_) (Figs. 3a,b), detects the presence of clear signals between 0.83 ppm and 3.40 ppm that explain cellulose modification by aliphatic chains (HDI-OH and HDI-OH-thiol). The peaks for (–NHCO–O–) carbamate group appear at 5.75 ppm for Cell-P_1_ and at 5.72 ppm for Cell-P_2_. Cellulose backbone signals appear between 3.60 and 3.90, while the methylene group (–CH_2_–) protons in the cellulose backbone give a signal at 4.35 and 4.29 ppm for Cell-P_1_ and Cell-P_2_, respectively. The ^1^H NMR spectra results are fitted with that reported by *El barkany *et al.^53^.

**Figure 4:**
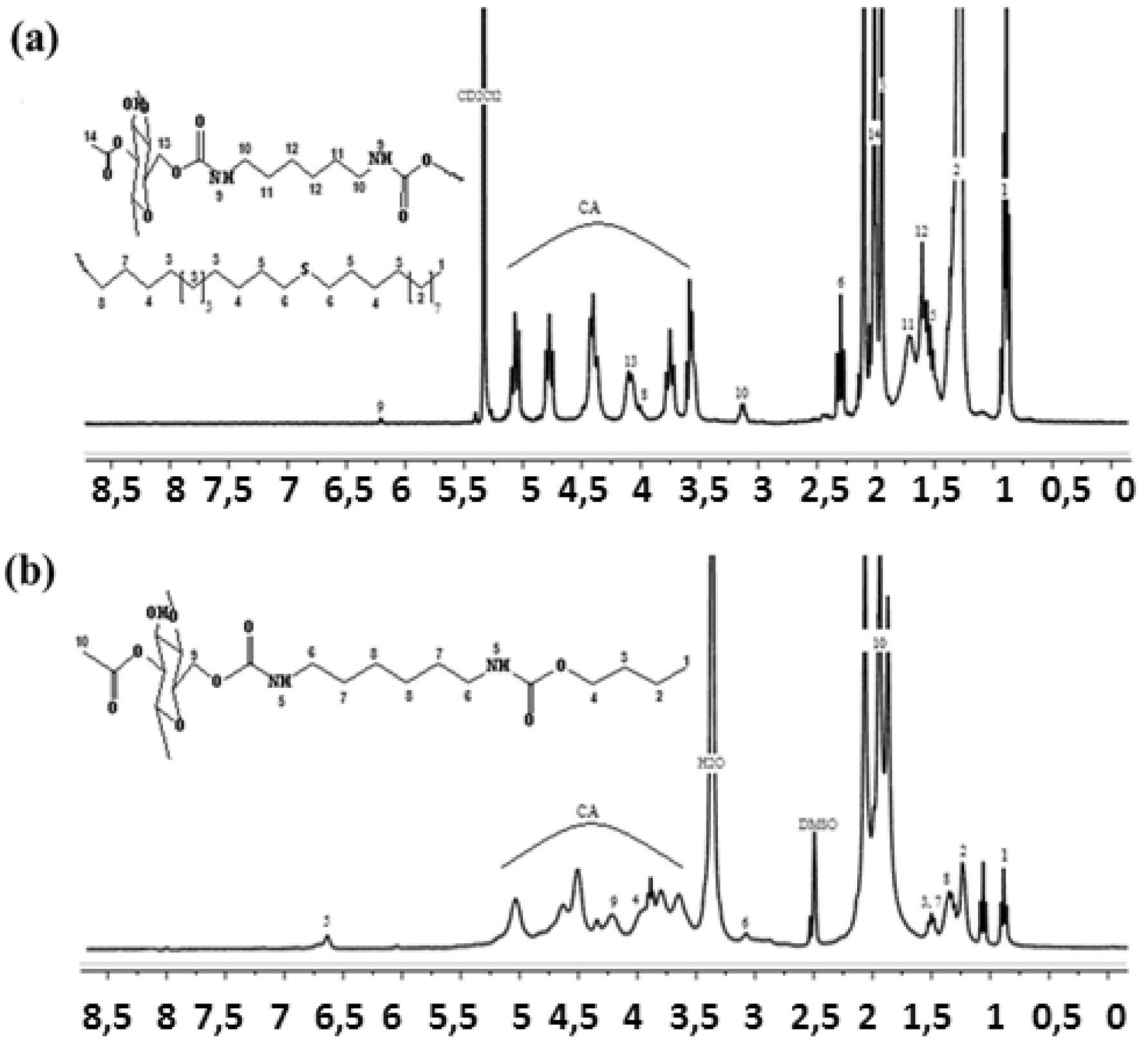
Proton NMR spectra for (**a**) CA-P_1_ and (**b**) CA-P_2_.

Figure 4 shows the Proton Nuclear Magnetic Resonance spectra for CA carbamate (CA-P_1_ and CA-P_2_). Such that, ^1^H NMR spectrum of CA-P_1_ (Fig. 4a) detects that methylene protons signals of the grafted chains appear between the values of 0.89 ppm and 3.74 ppm. The urethane group (–NHCO–O–) existence is confirmed by having a signal at 6.21 ppm. CA backbone protons are confirmed by appearance of signals between 3.56 ppm and 5.1 ppm, and the protons of acetyl group appear at 2.01 ppm. For CA-P_2_, ^1^H NMR spectrum shows a signal of CH_2_ between the values 0.89 ppm and 3.88 ppm (Fig. 4b). The presence of the urethane group (-NHCO-O-) is confirmed by a signal at value of 6.67 ppm. Finally, the protons of the cellulose acetate skeleton were justified by appearance of signals between 3.6 ppm and 5.1 ppm, and the protons of acetyl group are detected at 2.1 ppm. ^1^H NMR spectra results are fitted with that by Heinze et al.^54^.

**Figure 5:**
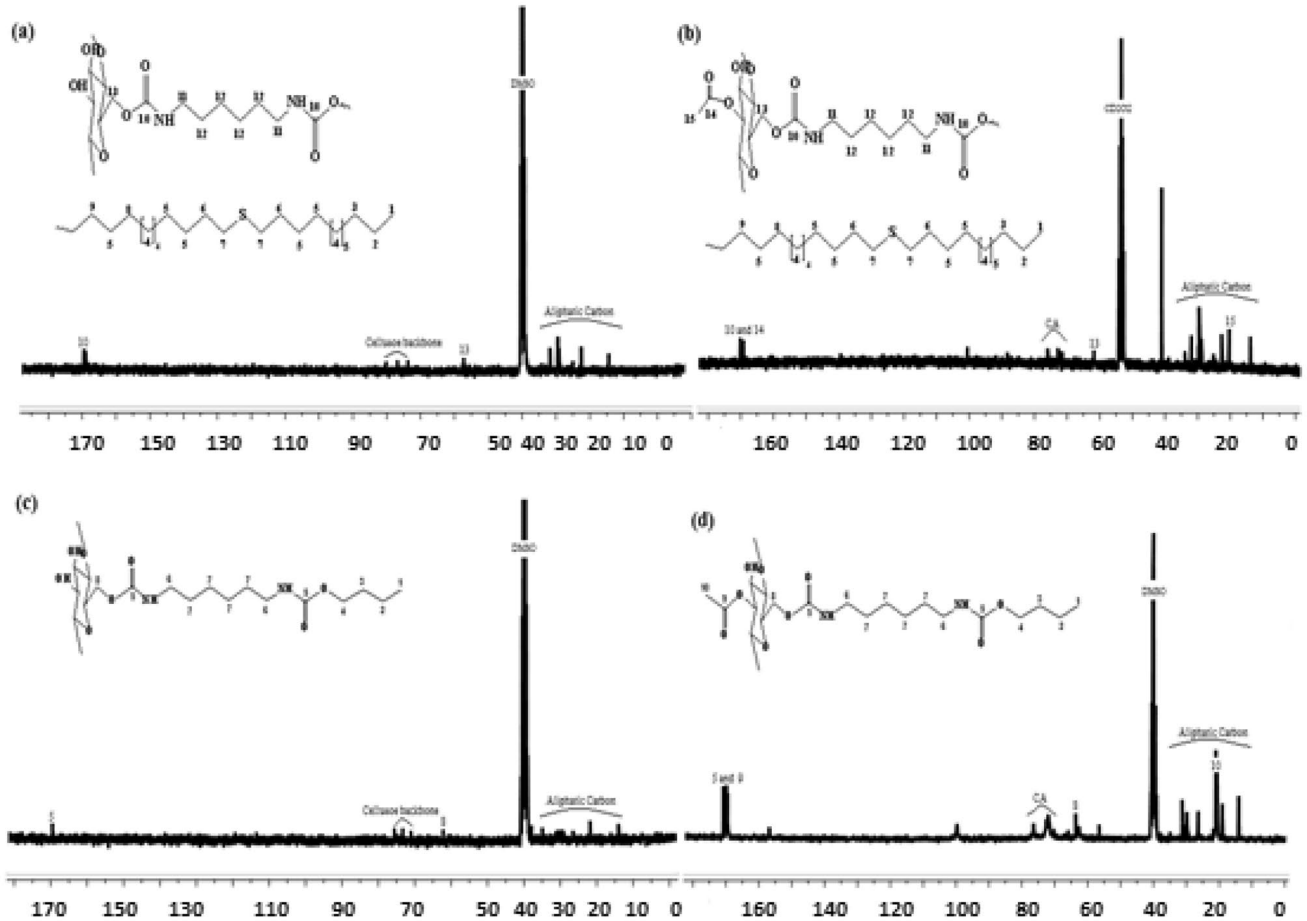
^13^C NMR spectra results for: (**a**) Cell-P_1_, (**b**) CA-P_1_, (**c**) Cell-P_2_, and (**d**) CA-P_2_.

The ^13^C NMR spectra results for the products (Cell-P_1_, Cell-P_2_, CA-P_1_, CA-P_2_) show signals that exist between 14 and 38 ppm, these signals are attributed to the presence of aliphatic carbons. This indicates forming of cellulose carbamate and CA carbamate (aliphatic chain grafted) (Fig. 5). Cellulose backbone signals appear between 76.6 ppm to 80 ppm, and from 70 to 77 ppm for Cell-P_1_ and Cell-P_2_, respectively. The peaks at 57.6 and 62.2 are fitted with the presence to methyl group (–CH_2_–) protons in the cellulose backbone for Cell-P_1_ and Cell-P_2_, respectively. In addition, carbonyl groups signals are typically appeared around value between 169.2 ppm and 170.3 ppm, corresponding respectively to the signals for carbonyl in ester group and carbonyl in urethane group (CA-P_1_). The carbonyl groups of CA-P_2_ give two signals around at 169.5 and 170.7 ppm corresponding, respectively, to the same groups already cited. Furthermore, the signals characterizing the urethane carbonyl groups of Cell-P_1_ and Cell-P_2_ appear at 169.1 and 169.4 ppm, respectively.

Proton Nuclear Magnetic Resonance technique was used to investigate the substitution (DS) degree of CA, cellulose carbamates and CA carbamates, using Eq. 3. The summarized results are shown in Table 2.^55^

4 $$DS=\frac{{n}_{AUG}*{I}_{i}}{{{n}_{i}*I}_{AUG}}$$

where ${I}_{i}$ represents the Integration for grafted-chains-protons (i); ${I}_{AUG}$ represents the Integration for cellulose-skeleton-protons; ${n}_{AUG}$ and ${n}_{i}$ represent proton numbers in CA or cellulose (${n}_{AUG}=7$), as well as, in the corresponding grafted group.

**Table 2:** Solubility tests for cellulose carbamate (Cell-P_1_ and Cell-P_2_), and cellulose acetate carbamate (CA-P_1_, CA-P_2_).

		Cell	Cell-P_1_ (DS = 0.8)	Cell-P_2_ (DS = 0.4)	CA (DS = 1.7)	CA-P_1_ (DS = 1.3)	CA-P_2_ (DS = 0.3)
Polar protic solvents	Ethanol	−	−	−	−	−	−
	Methanol	−	−	−	−	−	−
	Water	−	−	−	−	−	−
	Propane-2-ol	−	−	−	−	−	−
Polar aprotic solvents	DMSO	−	+	+	+	±	+
	DMF	−	±	±	+	±	+
	DMAc	−	±	±	±	±	±
	Acetone	−	−	−	−	−	−
	Acetonitrile	−	−	−	−	−	−
Non-polar aprotic solvents	Chloroform	−	−	±	−	−	±
	Dichloromethane	−	−	−	±	+	+
	Toluene	−	−	−	−	−	−
	Hexane	−	−	−	−	−	−
	THF	−	−	−	−	−	−
	Diethyl ether	−	−	−	−	−	−

+ : Soluble, ± : Partly soluble, −: insoluble.

### TGA analysis

**Figure 6:**
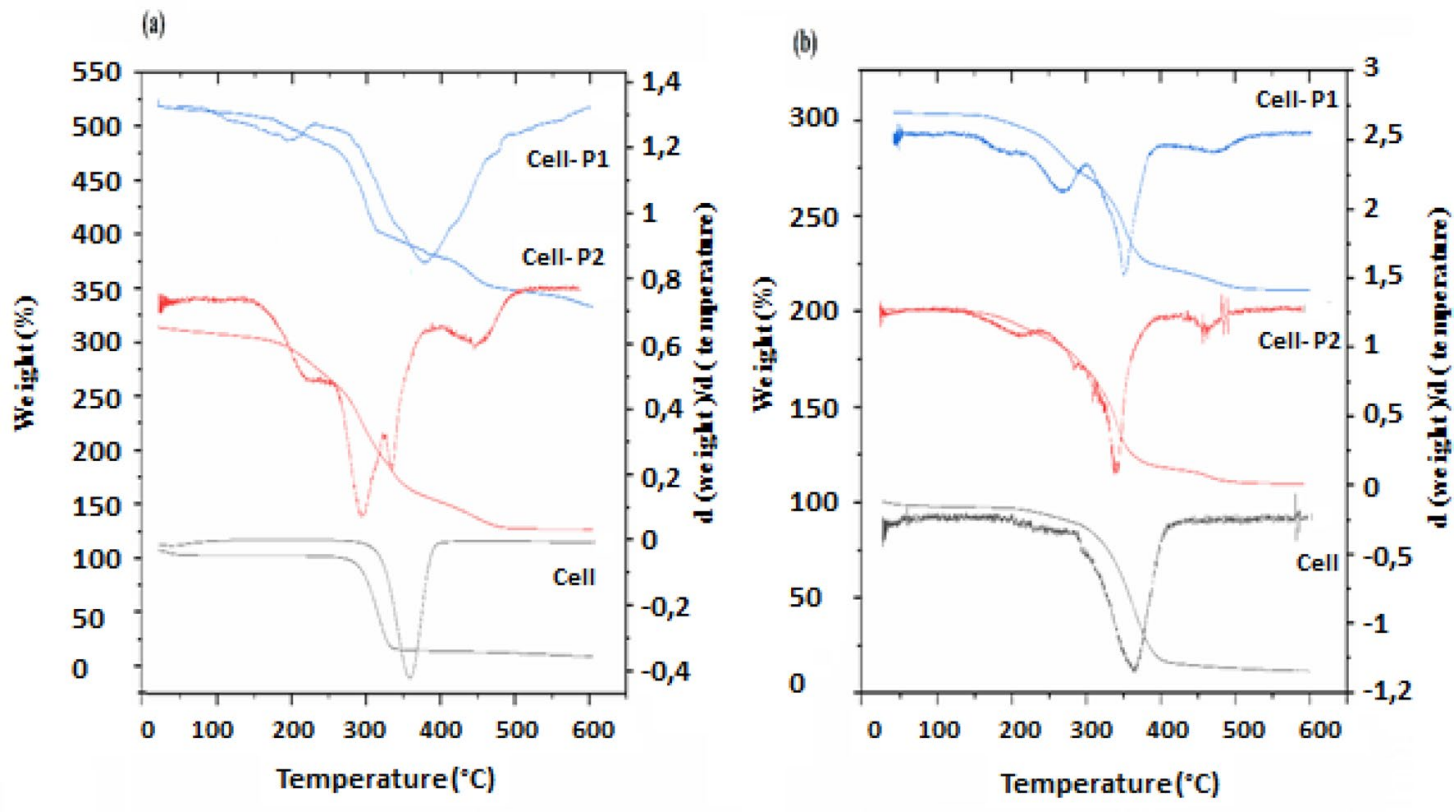
TGA/DTG thermograms for: (**a**) Cell-P_1_, Cell-P_2_, and cellulose, and (**b**) CA-P_1_, CA-P_2_, and CA.

TGA/DTG was used in order to investigate the thermal degradation behavior of the cellulose, CA, cellulose carbamate, and CA carbamate. The thermal stability for cellulose and the CA prior to and after modification has been compared (Fig. 6). From Fig. 6a, it can be found that cellulose shows a first weight loss at 20–100 °C, caused, probably, by the removal of residual solvents and moisture. The second loss, situated between 290 °C and 400 °C, due to cellulose-backbone decomposition. About 6% as percent of residual mass is comparable to the neat cellulose studied under the same conditions. It can be assumed that, as a result of the removal of volatile decomposition products, mainly char residues are formed. Others researchers confirmed the wide region of the weight loss of cellulose^49^.

Cell-P1 shows three steps of thermal degradation (Fig. 6a). The first step of weight loss is observed between temperatures of 130 °C and 263 °C. This step may be related to the decomposition of the grafted chain. The second step, located between 263 °C and 400 °C, is the thermal decomposition of the main chains in modified cellulose, similar to the decomposition of net cellulose. As shown in Fig. 6a, this process is accompanied by an intense endothermic thermal effect and a weight loss of 58%. The last step of thermal decomposition is observed between 400 °C and 530 °C, leaving only a negligible residue.

The thermal analysis of Cell-P2 identified also three steps of weight loss (Fig. 6a). Firstly, a step between 56 and 180 °C represents the elimination of solvents, particularly residual water. The second step, between 180 °C and 240 °C, relates to the decomposition of the grafted chains, while the last step above 240 °C is attributed to the decomposition of the main chains of cellulose derivative.

CA is decomposed into three steps (Fig. 6b). The first step begins at 26 and progresses to 200 °C, representing the volatilization of solvents and absorbed residual water. The second one is attributable to the degradation of CA main chains and ranges from 200 to 410 °C. The third step occurs after 410 °C and is may be caused by the products carbonization into an ash. The thermal degradation results for CA corroborate with that obtained by Senna et al*.*^56^.

The TGA/DTG curves (Figs. 6b) of CA-P_1_, illustrate three steps of weight loss. The modified CA begins to degrade at 120 °C and reaches a maximum temperature of around 340 °C. This maximum is shown in Fig. 6b with 39.5% of the mass degradation. The first step, located at temperature values between 120 °C and 250 °C, is due to the decomposition of the grafted groups on the CA. The second ranges from 250 °C to 420 °C and represents CA’s thermal decomposition. After 420 °C, the last step is observed. In addition, for CA-P_2_, the thermal analysis detects three slopes of mass degradation. The step between 40 °C and 210 °C involves solvents removal, especially residual water. The first mass loss, that is located at temperatures between 210 °C and 300 °C, shows the decomposition of the grafted groups on the CA. The second step, located between 300 °C and 400 °C, is a weight loss caused by the decomposition of CA backbone. The last step starts at 400 °C to 600 °C. Then, we can deduce that cellulose carbamates and CA carbamates are less stable than their primer analogues. This reduction in thermal stability might be attributed to the high substitution degree (DS ~ 3) of these derivatives, which eliminates the interactions that existed between the cellulosic chains previously.

### Solubility test

Dissolution tests were carried out for cellulose acetate carbamate and cellulose carbamate compounds. The procedure for these tests includes the following: Firstly, dried samples were placed in a suitable weighting bottle, and then dried at 70 °C in a vacuum oven. Secondly, dried samples were placed in a bottle at room temperature with an adequate solvent for carbamate of cellulose acetate and under heating for other samples, the mixture was kept under stirring for enough time until the powder disappears depending on visual monitoring). The solubility of CA carbamates and cellulose carbamates in several solvent systems are shown in Table 2. The solubility values of cellulose and CA are supposed to be known.

In general, the introduction of some groups, for example, carbamate groups into cellulose and CA, changes their solubility behaviors. The obtained derivatives are soluble in DMSO and partly soluble in DMF and DMAc at room temperature. Cell-P_2_ and CA-P_2_ are partly soluble in chloroform at room temperature. Moreover, CA carbamates are usually soluble in CH_2_Cl_2_ at room temperature. In summary, the transformation of cellulose and CA into carbamates leads to improved solubility because the carbamates become hydrophobic and exhibit a good solubility phenomenon in polar aprotic solvent systems, as well as, in the non-polar aprotic solvents (chloroform and dichloromethane) at room temperature.

### X-ray diffraction

**Figure 7:**
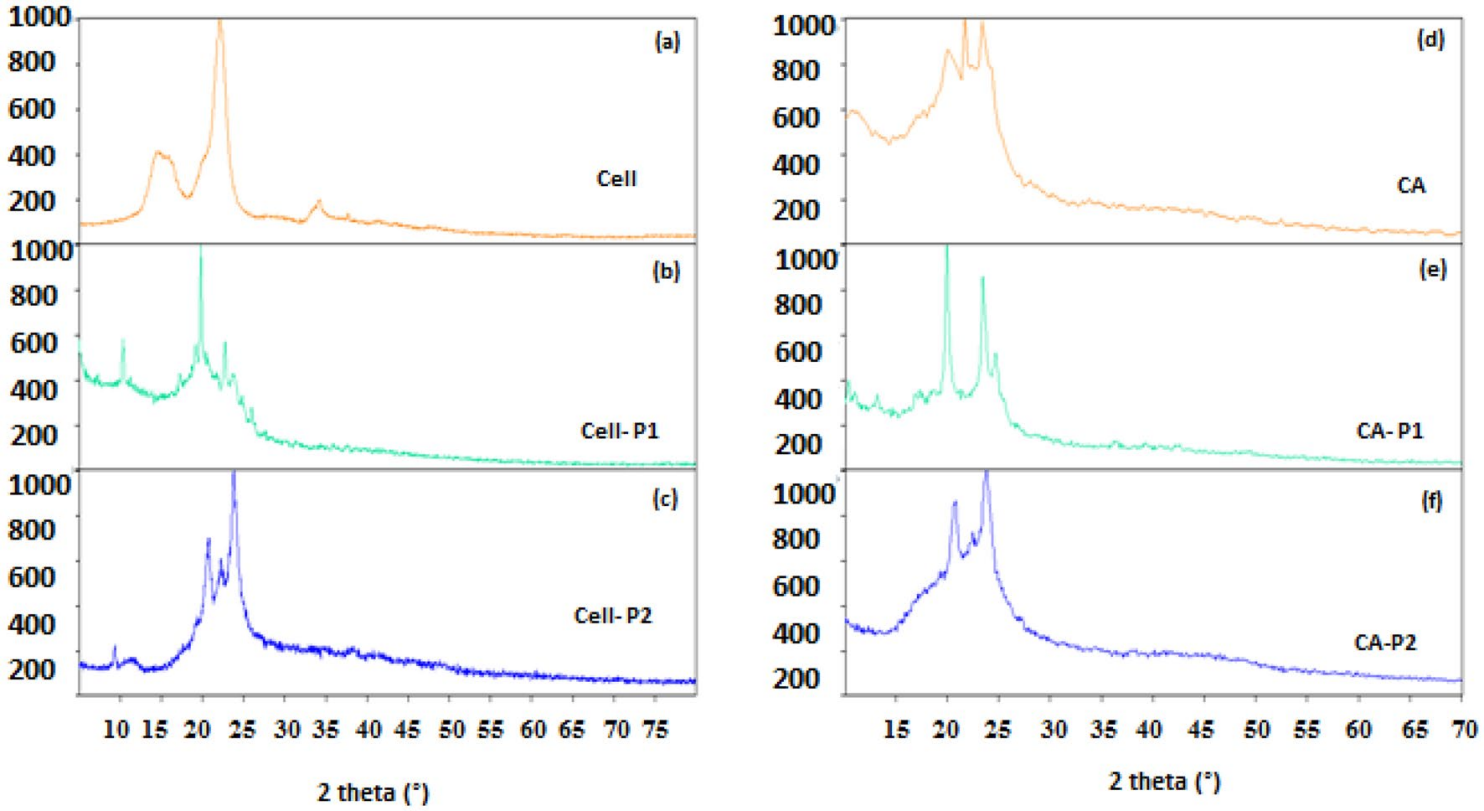
XRD spectra for (**a**) cellulose, (**b**) Cell-P_1_, (**c**) Cell-P_2_, (**d**) CA, (**e**) CA-P_1_, and (**f**) CA-P_2._

The study of X-ray diffraction provides to obtain data on changes in the supramolecular structure of cellulose and CA as a result of their grafting. The X-ray diffractogram of cellulose (Fig. 7a) has reflections in the 2θ regions of 14, 15, 22 and 34 degrees attributed to 11 ®0, 110, 200, and 400 planes that is typical of the CI allomorph^44^. As can be seen from Fig. 7b,c, after the formation of cellulose carbamates, typical reflections, characteristic of the original cellulose, disappear and new reflections appear. In addition, the structure of the obtained carbamates becomes more disordered.

Regarding sample of AC, more precisely, diacetate cellulose, it is known that it has an amorphous structure. X-ray reflections for this sample can be associated with the presence of the near order in the structure of the amorphous polymer. After the formation of carbamates, the X-ray diffraction pattern of the original CA changes significantly.

Thus, the grafting process with the formation of carbamates caused a clear change in the supramolecular structure of the initial polymers, both cellulose and AC.

The low degree of structural order, 64%, for acetylated cellulose compared to cellulose, 88% (Table 3), is a result of the substitution of hydroxyl groups by the acetyl groups having a larger volume and breaking the intermolecular and intramolecular hydrogen bonds in cellulose^57^. The "OI" value decreases from 88% for cellulose to 59% for Cell-P1 and 76% for Cell-P2, showing a significant reduction in structural order, and ease of transformation^58^. These changes may also be related to differences in the degree of grafting (DS). In addition, this indicates that as a result of the modification of cellulose, the final products became less ordered.

**Table 3:** Ordering index (OI) for cellulose and its derivatives.

Samples	Ordering index (OI (%))
Cellulose	88.63
Cell-P_1_	59.12
Cell-P_2_	76.59
CA	63.98
CA-P_1_	81.64
CA-P_2_	69.32

The ordering index of CA carbamates varies from 69 to 81%. Thus, the structural order of CA derivatives can depend to a large extent on the reaction conditions, the DS value, and the chemical structure of the grafted molecules.

### Biodegradability

**Figure 8:**
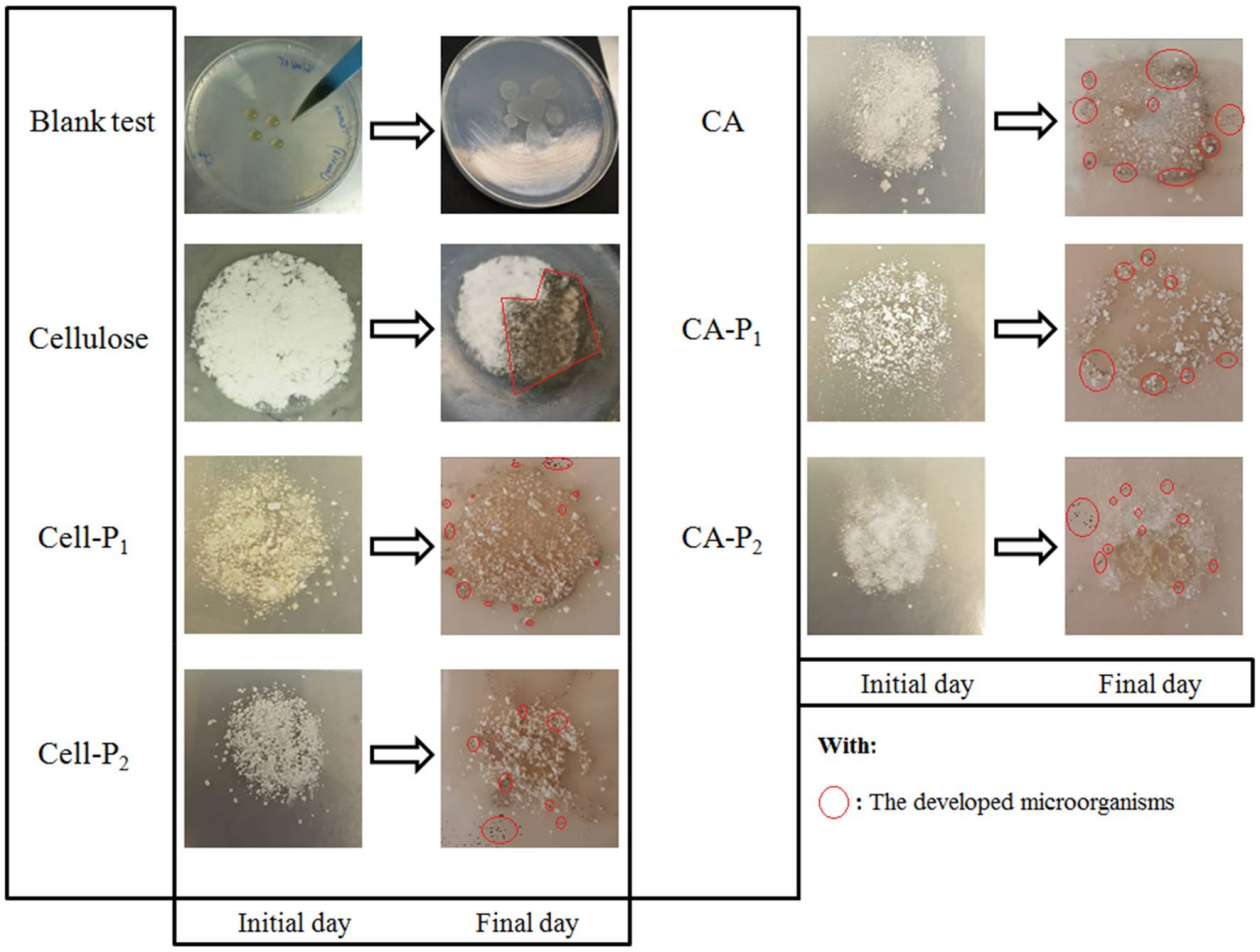
Biodegradation evolution of cellulose, Cell-P_1_, Cell-P_2_, CA, CA-P_1_, and CA-P_2_ after 28 days of incubation under aerobic conditions on culture medium.

The biodegradability of the samples (Cell, Cell-P1, Cell-P2, CA, CA-P1, and CA-P2) was determined under aerobic liquid and culture medium conditions according to ASTM (G21-70, G22-76, and G29-75), DIN 53739, AFNOR (X 41-514-81 and X41-517-69), ISO 14,851 and ISO 846 methods. In these experiments, the samples studied were used as the sole carbon sources in the medium. The experiments for aerobic biodegradation in the culture medium conditions were detected by a visual evaluation, noting that the amount of microbial growth on the material surface or in clear areas was due to substrate hydrolysis by the enzymes released by microorganisms. Figure 8 summarized the biodegradation evaluation results of the blank test (absence of biopolymer), cellulose, Cell-P_1_, Cell-P_2_, CA, CA-P_1_, and CA-P_2_ samples under aerobic conditions on culture medium. After an incubation period of 28 days, it was noticed visually that colonization of cellulose compound and cellulose derivatives occurred with variable incidence by microorganisms associated with the lixivia that contain a high and varied microbial load. . On the other hand, microbial growth was not observed at all, in the case of a blank test (i.e. absence of samples). These results clearly indicate the biodegradation of the samples, which are the only source of carbon for the growing microorganisms.

The aerobic biodegradation of the samples in liquid conditions was evaluated by measuring the biochemical oxygen demand (BOD) in a closed respirometer. The values of biodegradability were measured as the amount of oxygen consumed in mg during biodegradation of the biopolymer per volume of culture-medium in Liter (biodegradation unit: mg O_2_/L). The amount of oxygen consumed in the biodegradation experiments for the cellulose derivatives (after blank-test correction) was represented as the theoretical oxygen demand (ThOD) percentage. ThOD is measured as oxygen mass per biopolymer mass, which is determined by finding the oxygen amount necessary for the aerobic biodegradation of all samples (i.e., the complete oxidation of the carbon into carbon dioxide).

**Figure 9:**
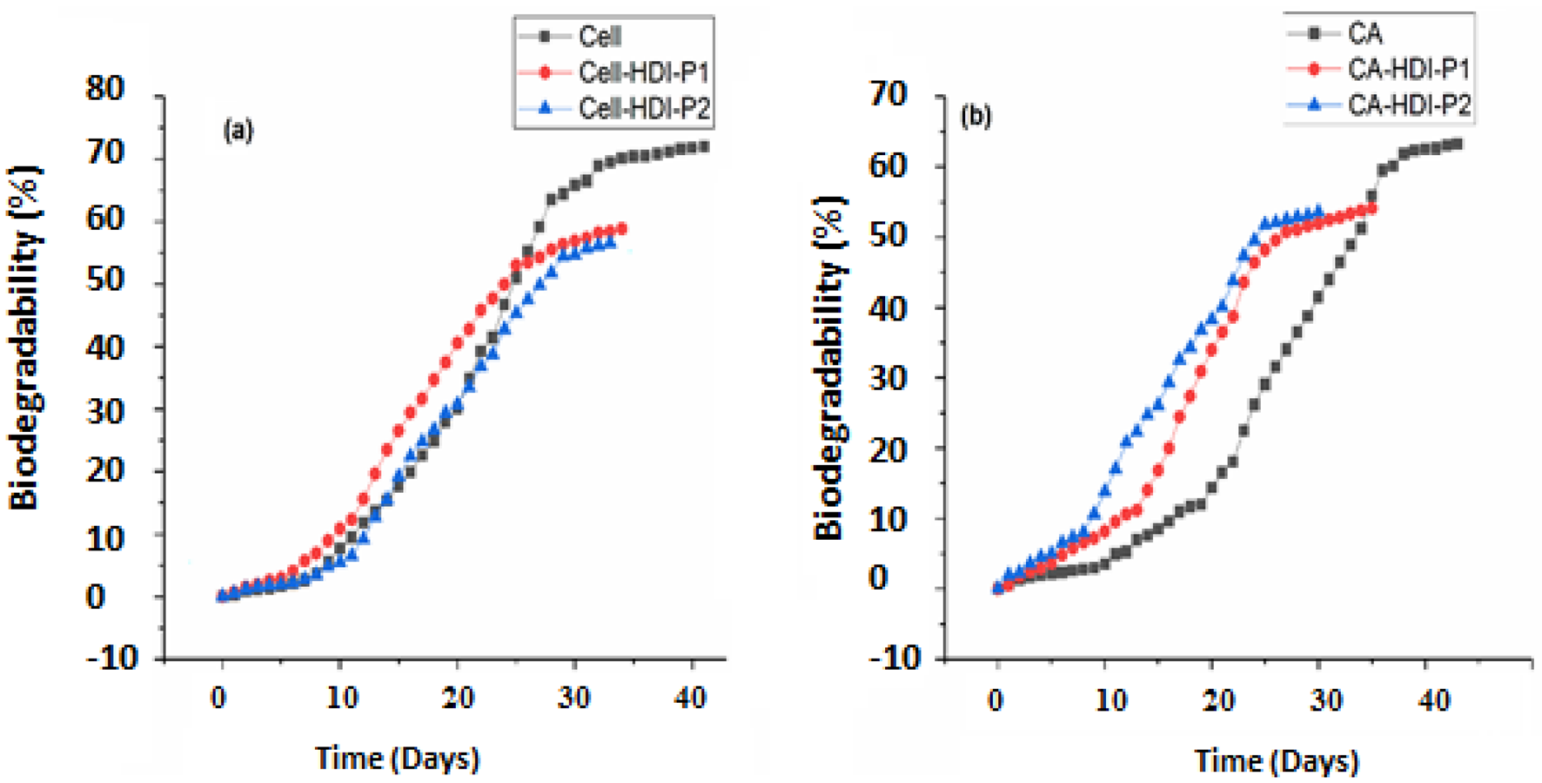
Biodegradation percentage of: (**a**) cellulose, Cell-P_1_, and Cell-P_2_, and (**b**) CA, CA-P_1_, and CA-P_2_ at 25 °C under aerobic liquid conditions after incubation period of 40 days.

The results of the biodegradation (%) obtained during 40 days of culture at 25 °C for the biopolymer samples studied are indicated in Fig. 9. It is important to notice that the curves of biodegradation (BD) of all samples are detected in the first phase (approximately 8 days), characterized by a low increase in the value of BD. This phase may be due to the microorganisms’ adaptation to the new environment by secreting the enzymes that are necessary for the new carbon sources’ biodegradation, and it could be assimilated to the lag-phase of the microbial growth.

The second phase of the curves of biodegradation (BD) is characterized by BD values that are increasing exponentially for all samples. This phase may be assimilated to the microorganisms’ exponential growth phase and detect the quick and high biodegradation process for biopolymers by the microorganisms associated with the soil. In general, it is modeled using the following exponential equation:

5 $$BD={BD}_{0}{e}^{\mu t}$$

6 $$\mathit{ln}BD=\mu (t-{t}_{0})+\mathit{ln}{BD}_{0}$$

where BD: biodegradation value at t, BD_0_: biodegradation value at t_0_, and μ: biodegradation rate.

The third phase, located in the curves of biodegradation, is characterized by a low increase in the values of BD for all samples. This phase could be assimilated to the stationary phase of microbial growth, as well as, it could be a result of the nutrient low concentration, especially the carbon sources (biopolymers).

The obtained data of BOD were measured as the theoretical total biodegradation percentage (Fig. 9). The experimental results show that after an incubation period of 40 days (Fig. 9a), the cellulose is biodegraded over 70% and the Cell-P_1_ is biodegraded over 59% after an incubation period of 34 days. After 33 days of incubation, Cell-P2 has been biodegraded by 56%. Additionally, CA is biodegraded over 62% after 40 days of incubation, CA-P_1_ is biodegraded over 54% after an incubation period of 35 days, and CA-P_2_ is biodegraded over 53% after an incubation period of 30 days (Fig. 9b). The experimental results concluded that cellulose is more biodegradable than its derivatives because it possesses high wettability, and CA is more biodegradable than its counterpart derivatives. By comparing these results with those in Table 4, it can be seen that Cell-P_1_ and CA-P_2_ are more biodegradable than the other derivatives. The introduction of carboxyl groups increases the tendency of the samples to degrade, but the introduction of carbamates acts negatively and hinders the biodegradation process because carbamate groups are more stable and therefore harder to break down. Thus cellulose carbamate and CA carbamate have a low percentage of biodegradation that does not exceed 58%. According to Table 4, we can see that the biodegradation rate has the following order: Cell-P_2_ $<$ Cellulose $<$ Cell-P_1_ and CA-P_2_ $<$ CA $<$ CA-P_1_. Hence, the introduction of the longer alcohol (undec-10-enol) in CA and cellulose significantly increased their biodegradation rates. On the other hand, the incorporation of the short alcohol (butanol) in CA and cellulose slightly reduced their biodegradation rates. Such that, the increase in biodegradation rate of the biopolymer samples could be explained by the existence of more ester bonds in Cell-P_1_, and CA-P_1_ than in Cell, CA, Cell-P_2,_ and CA-P_2_, where the microbial attack could happen by esterase-enzymes existence. Fungi that are associated with the biopolymers samples, are known for their capacity of esterase production on different plant polysaccharides^59^.

**Table 4:** Rate of biodegradation of tested biopolymers.

Biopolymer	Rate of biodegradation (μ)
Cell	0.11
Cell-P_1_	0.15
Cell-P_2_	0.10
CA	0.12
CA-P_1_	0.14
CA-P_2_	0.11

In general, it is detected that soil and lixivia can be used as inoculum, having a promising effect on biodegradation, and that the microorganisms’ growth is due to their ability to use these biopolymer samples as sources of carbon. The results that are investigated by the method of BOD are mostly in line with the results detected by the microbial-growth method. Cell-P_1_ and CA-P_1_ are the fastest biodegradable samples.

## Conclusion

A novel modification process was done in order to develop cellulose carbamate and cellulose acetate carbamate. In the first stage, a blocked isocyanate (precursor) is prepared from thiol-ene addition of undec-10-enol with dodecanthiol, which is then added to 1,6-HDI or butanol addition to 1,6-HDI. In a subsequent stage, the precursor is reacted with cellulose and CA in a homogenous medium via an addition reaction. In comparison to existing traditional methods, the procedure is considered cost-effective, pollution-free, easy to scale-up without by-products, and conducted in one-pot. NMR, FT-IR, and XRD analyses, as well as solubility and biodegradation studies, were used to confirm the structures, solubility, and biodegradability of the biopolymers produced. Our results showed that the carbamate groups are successfully introduced onto the cellulose and CA backbones and that these biopolymers, namely Cell-P_1_ and CA-P_1_, are more biodegradable and soluble than their original raw material (cellulose). Indeed, they may be used to replace non-biodegradable polymers in various industrial fields, such as; eco-friendly food packaging, domains that use materials that are environmentally friendly and sustainable, and the development of green chemistry.

## Data Availability

The datasets used and/or analyzed during the current study are available from the corresponding author on reasonable request.

## References

[1] Jawaid M, Abdul Khalil HPS (2011). Cellulosic/synthetic fibre reinforced polymer hybrid composites: A review. Carbohydr. Polym..

[2] Qiu X, Tao S, Ren X, Hu S (2012). Modified cellulose films with controlled permeatability and biodegradability by crosslinking with toluene diisocyanate under homogeneous conditions. Carbohydr. Polym..

[3] Bernardo P, Drioli E, Golemme G (2009). Membrane gas separation: A review/state of the art. Ind. Eng. Chem. Res..

[4] Ahmad AL, Jawad ZA, Low SC, Zein SHS (2014). A cellulose acetate/multi-walled carbon nanotube mixed matrix membrane for CO2/N2 separation. J. Memb. Sci..

[5] Azzaoui K (2014). Synthesis and characterization of composite based on cellulose acetate and hydroxyapatite application to the absorption of harmful substances. Carbohydr. Polym..

[6] Shokri, J. & Adibkia, K. Application of Cellulose and Cellulose Derivatives in Pharmaceutical Industries. in *Cellulose – Medical, Pharmaceutical and Electronic Applications* (ed. Ven, T. G. M. Van De) 47–66 (2013).

[7] Frohoff-Hülsmann MA, Lippold BC, McGinity JW (1999). Aqueous ethyl cellulose dispersion containing plasticizers of different water solubility and hydroxypropyl methyl-cellulose as coating material for diffusion pellets II: Properties of sprayed films. Eur. J. Pharm. Biopharm..

[8] Frohoff-Hu MA, Lsmann AS, Lippold BC (1999). Aqueous ethyl cellulose dispersions containing plasticizers of different water solubility and hydroxypropyl methylcellulose as coating material for diffusion pellets I. Drug release rates from coated pellets. Int. J. Pharm..

[9] Hokkanen S, Repo E, Sillanpää M (2013). Removal of heavy metals from aqueous solutions by succinic anhydride modified mercerized nanocellulose. Chem. Eng. J..

[10] Zhou Y, Jin Q, Hu X (2012). Heavy metal ions and organic dyes removal from water by cellulose modified with maleic anhydride. J. Master Sci..

[11] Anirudhan TS, Nima J, Divya PL (2013). Adsorption of chromium(VI) from aqueous solutions by glycidylmethacrylate- grafted-densified cellulose with quaternary ammonium groups. Appl. Surf. Sci..

[12] Azzaoui K (2017). Preparation and characterization of biodegradable nanocomposites derived from carboxymethyl cellulose and hydroxyapatite. Carbohydr. Polym..

[13] Baptista, A., Ferreira, I. & Borges, J. Cellulose-Based Bioelectronic Devices. in *Cellulose - Medical, Pharmaceutical and Electronic Applications* (ed. Ven, T. G. M. Van De) 67–82 (2013). 10.5772/56721.

[14] Yu G, Teng Y, Lai W, Yin C (2016). The preparation and study of cellulose carbamates and their regenerated membranes. Int. J. Biol. Macromol..

[15] Gandini, A. & Belgacem, M. N. Modifying cellulose fiber surfaces in the manufacture of natural fiber composites. in *Interface engineering of natural fibre composites for maximum performance* 3–42 (Woodhead Publishing Limited, 2011). doi:10.1533/9780857092281.1.3.

[16] Azizi Samir MAS, Alloin F, Dufresne A (2005). Review of recent research into cellulosic whiskers, their properties and their application in nanocomposite field. Biomacromol.

[17] Rueda L (2011). Isocyanate-rich cellulose nanocrystals and their selective insertion in elastomeric polyurethane. Compos. Sci. Technol..

[18] Ashori A, Babaee M, Jonoobi M, Hamzeh Y (2014). Solvent-free acetylation of cellulose nanofibers for improving compatibility and dispersion. Carbohydr. Polym..

[19] Heinze, T., Seoud, O. A. El & Koschella, A. Etherification of Cellulose: Synthesis, Structure, and Properties. in *Cellulose Derivatives* (ed. Thomas Heinze, Omar A. El Seoud, A. K.) 429–477 (2018).

[20] Han YD, Yang PF, Li JY, Qiao CD, Li TD (2010). The reaction of o-hydroxybenzyl alcohol with phenyl isocyanate in polar solvents. React. Kinet. Mech. Catal..

[21] Siqueira G, Bras J, Dufresne A (2010). New process of chemical grafting of cellulose nanoparticles with a long chain isocyanate. Langmuir.

[22] Khan FZ, Shiotsuki M, Nishio Y, Masuda T (2008). Synthesis, characterization, and gas permeation properties of t-butylcarbamates of cellulose derivatives. J. Memb. Sci..

[23] Diamantoglou M, Platz J, Vienken J (1999). Cellulose carbamates and derivatives as hemocompatible membrane materials for hemodialysis. Artif. Organs.

[24] Gironès J, Pimenta MTB, Vilaseca F, Carvalho AJF, Mutje P (2008). Blocked diisocyanates as reactive coupling agents : Application to pine fiber—polypropylene composites. Carbohydr. Polym..

[25] Oliveros L, Senso A, Franco P, Minguillón C (1998). Carbamates of cellulose bonded on silica gel: Chiral discrimination ability as HPLC chiral stationary phases. Chirality.

[26] Paunonen S (2019). Environmental impact of cellulose carbamate fibers from chemically recycled cotton. J. Clean. Prod..

[27] Zhang Y, Li H, Li X, Gibril ME, Yu M (2014). Chemical modification of cellulose by in situ reactive extrusion in ionic liquid. Carbohydr. Polym..

[28] Gan S, Zakaria S, Chia CH, Kaco H, Padzil FNM (2014). Synthesis of kenaf cellulose carbamate using microwave irradiation for preparation of cellulose membrane. Carbohydr. Polym..

[29] Fu F (2015). Improved synthesis of cellulose carbamates with minimum urea based on an easy scale-up method. ACS Sustain. Chem. Eng..

[30] Pinnow M, Fink H-P, Fanter C, Kunze J (2008). Characterization of highly porous materials from cellulose carbamate. Macromol. Symp..

[31] Selin J-F (1985). Method of producing cellulose carbamate fibers or films. System.

[32] Xu M (2020). Preparation and characterization of cellulose carbamate membrane with high strength and transparency. J. Appl. Polym. Sci..

[33] Willberg-Keyriläinen P, Hiltunen J, Ropponen J (2017). Production of cellulose carbamate using urea-based deep eutectic solvents. Cellulose.

[34] Guo Y, Zhou J, Song Y, Zhang L (2009). An efficient and environmentally friendly method for the synthesis of cellulose carbamate by microwave heating. Macromol. Rapid Commun..

[35] Guo Y, Zhou J, Wang Y, Zhang L, Lin X (2010). An efficient transformation of cellulose into cellulose carbamates assisted by microwave irradiation. Cellulose.

[36] Thi LTV, Siroká B, Manian AP, Bechtold T (2010). Functionalisation of cellulosic substrates by a facile solventless method of introducing carbamate groups. Carbohydr. Polym..

[37] Zhang Y, Yin C, Zhang Y, Wu H (2013). Synthesis and characterization of cellulose carbamate from wood pulp assisted by supercritical carbon dioxide. BioResources.

[38] Iller E, Stupinska H, Starostk P (2007). Properties of cellulose derivatives produced from radiation-Modified cellulose pulps. Radiat. Phys. Chem..

[39] Yin C (2007). Chemical modification of cotton cellulose in supercritical carbon dioxide: Synthesis and characterization of cellulose carbamate. Carbohydr. Polym..

[40] Yin C, Shen X (2007). Synthesis of cellulose carbamate by supercritical CO2-assisted impregnation: Structure and rheological properties. Eur. Polym. J..

[41] Wang X, Wu HY, Yin CY (2013). Preparation and characterization of cellulose carbamate regeneration membrane by supercritical carbon dioxide. Adv. Mater. Res..

[42] Xiong LK, Yu GM, Yin CY (2017). Synthesis and characterization of cellulose carbamate by liquid-solid phase method. Fibers Polym..

[43] El Idrissi A, El Barkany S, Amhamdi H, Maaroufi AK (2013). Physicochemical characterization of celluloses extracted from Esparto ‘stipa tenacissima’ of Eastern Morocco. J. Appl. Polym. Sci..

[44] Segal L, Creely JJ, Martin AE, Conrad CM (1959). An Empirical Method for Estimating the Degree of Crystallinity of Native Cellulose Using the X-Ray Diffractometer. Text. Res. J..

[45] Doyle S, Pethrick RA (1987). Structure of fibrous cellulose acetate: X-ray diffraction, positron annihilation and electron microscopy investigations. J. Appl. Polym. Sci..

[46] Calmon-Decriaud A, Bellon-Maurel V, Frangoise S (1998). Standard methods for testing the aerobic biodegrad ion of polymeric materials. Rev. Petsives. Adv. Polym. Sci..

[47] Krzan A, Hemjinda S, Miertus S, Corti A, Chiellini E (2006). Standardization and certification in the area of environmentally degradable plastics. Polym. Degrad. Stab..

[48] Boutevin B (2000). From telomerization to living radical polymerization. J. Polym. Sci. Part A Polym. Chem..

[49] Gupta VK, Carrott PJM, Singh R, Chaudhary M, Kushwaha S (2016). Cellulose: A review as natural, modified and activated carbon adsorbent. Bioresour. Technol..

[50] Belgacem, M. N. & Gandini, A. *Monomers, Polymers and Composites from Renewable Resources*. (2008).

[51] Gironès J (2007). Blocked isocyanates as coupling agents for cellulose-based composites. Carbohydr. Polym..

[52] Gallego R, Arteaga JF, Valencia C, Franco JM (2013). Rheology and thermal degradation of isocyanate-functionalized methyl cellulose-based oleogels. Carbohydr. Polym..

[53] El Barkany S (2016). Homogeneous grafting of new amido groups onto hydroxyethyl cellulose acetate microfibrils: Solubility study. Moroccan J. Chem..

[54] Heinze T, Liebert T (2004). Chemical characteristics of cellulose acetate. Macromol. Symp..

[55] Jandura PV, Kokta B, Riedl B (2000). Fibrous long-chain organic acid cellulose esters and their characterization by diffuse reflectance FTIR spectroscopy, solid-state CP/MAS 13 C-NMR, and X-ray diffraction. J. Appl. Polym. Sci..

[56] Senna AM, Monteiro K, Botaro VR (2014). Synthesis and characterization of hydrogels from cellulose acetate by esterification crosslinking with EDTA dianhydride. Carbohydr. Polym..

[57] Hu W, Chen S, Xu Q, Wang H (2011). Solvent-free acetylation of bacterial cellulose under moderate conditions. Carbohydr. Polym..

[58] El Idrissi A, El Barkany S, Amhamdi H, Maaroufi AK (2012). Synthesis and characterization of the new cellulose derivative films based on the hydroxyethyl cellulose prepared from esparto ‘stipa tenacissima’ cellulose of Eastern Morocco. II. Esterification with acyl chlorides in a homogeneous medium. J. Appl. Polym. Sci..

[59] Biely P (2012). Microbial carbohydrate esterases deacetylating plant polysaccharides. Biotechnol. Adv..

